# Metformin Alleviates Cadmium-Induced Autophagic Flux Impairment-Dependent Apoptosis by Activating AMPK in Neuronal Cells

**DOI:** 10.3390/cells15080739

**Published:** 2026-04-21

**Authors:** Wen Wu, Xiaoling Chen, Tong Ji, Qianyun Yu, Long Hou, Zhihan Zhou, Baoming Gong, Ming Xu, Wei Gao, Shile Huang, Long Chen

**Affiliations:** 1 Jiangsu Key Laboratory for Molecular and Medical Biotechnology, College of Life Sciences, Nanjing Normal University, Nanjing 210023, China; wen1.wu@legendbiotech.cn (W.W.); xlchen@wnmc.edu.cn (X.C.); jitongjuxie@126.com (T.J.); yqy1316106010@163.com (Q.Y.); larry.hou@genuinbiotech.cn (L.H.); 18362981813@163.com (Z.Z.); 22110700022@m.fudan.edu.cn (B.G.); mia.xu@genuinbiotech.cn (M.X.); gaowei@njnu.edu.cn (W.G.); 2 Department of Blood Transfusion, The First Affiliated Hospital of Wannan Medical College, Wuhu 241001, China; 3 Department of Biochemistry and Molecular Biology, Louisiana State University Health Sciences Center, Shreveport, LA 71103, USA; 4 Department of Hematology and Oncology, Louisiana State University Health Sciences Center, Shreveport, LA 71103, USA; 5 Feist-Weiller Cancer Center, Louisiana State University Health Sciences Center, Shreveport, LA 71103, USA

**Keywords:** metformin, cadmium, ATG5, autophagic flux, AMPK, neuronal cells

## Abstract

Cadmium (Cd), a common environmental and occupational toxicant, is an important risk factor for neurodegenerative diseases. Metformin has been found to have neuroprotective effect, in addition to antidiabetic function. Our recent studies have identified that metformin ameliorates Cd neurotoxicity via blocking ROS-dependent PP5/AMPK-JNK signaling pathway. Here we further show that metformin protected PC12 cells and primary neurons from Cd-poisoning by mitigating Cd-induced increases in ATG5/LC3-II/p62 levels and autophagosomes. Knockdown of ATG5 dramatically potentiated the inhibitory effects of metformin on Cd-induced LC3-II, cleavage of caspase-3, accumulation of autophagosomes and apoptosis in PC12 cells. Addition of chloroquine (CQ) strengthened the basic and Cd-elevated ATG5/LC3-II/p62 levels, autophagosome accumulation and cell apoptosis, whereas metformin powerfully blocked the events, implying a metformin-promoted autophagic flux-dependent mechanism involved. Further research revealed that metformin prevented Cd-induced autophagic flux impairment and cell apoptosis, which was attributed to restraining Cd inactivation of AMPK. This is supported by the findings that activation of AMPK with AICAR or ectopic expression of constitutively active AMPKα (AMPKα-ca) reinforced the inhibitory effects of metformin on Cd-evoked ATG5/LC3-II/p62/autophagosomes and apoptosis in PC12 cells and/or primary neurons. Taken together, the results indicate that metformin protects neuronal cells from Cd-induced autophagic flux impairment-dependent apoptosis by activating AMPK. Our studies highlight that metformin has a great potential for prevention of Cd toxicity related to neurodegenerative diseases.

## 1. Introduction

Cadmium (Cd), a well-known pollutant that is hazardous to human health, is a metal element of Earth’s crust that is widely spread in the environment, primarily due to industrial and agricultural activities [1]. Drinking water, cigarette smoking, recharged nickel–cadmium batteries, and foods (e.g., cereals, vegetables, potatoes, and meat products) are the major sources of human Cd exposure [1,2]. Cd has high residence and low clearance in a variety of organs, and the half-life of Cd in humans is up to 10–30 years [3]. Cd toxicity results in a variety of health problems, such as cancer, kidney disease, osteoporosis, atherosclerosis, and cardiovascular diseases [4,5]. Among the various Cd-related diseases, the neurotoxicity has attracted great attention in recent years [3,6,7,8]. After being absorbed into the bloodstream, Cd can destroy the structural integrity of the blood–brain barrier, allowing Cd to enter and accumulate in the brain [9,10,11]. Exposure to Cd can severely affect the functions of the nervous system with symptoms including headache and vertigo, olfactory dysfunction, slow vasomotor function, peripheral neuropathy, decreased equilibrium, decreased ability to concentrate, and learning disabilities, which may eventually lead to consequential neurodegenerative diseases including Alzheimer’s disease (AD), Parkinson’s disease (PD), Huntington’s disease (HD), and Amyotrophic Lateral Sclerosis (ALS) [3,6,12,13,14].

Autophagy, an evolutionarily conserved cellular process, has been considered as a homeostatic, catabolic degradation process to preserve cellular function [15,16]. Emerging evidence suggests that autophagy dysfunction contributes to several diseases, including neurodegenerative diseases [17,18,19]. One of the hallmarks of neurodegenerative diseases is the accumulation of misfolded proteins and damaged organelles in and around neurons, and hence, disease severity is thought to be highly dependent on autophagic activity within neurons, as well as non-neuronal cells [20,21,22,23]. Lipidation of the microtubule-associated protein light chain 3-I (LC3-I) to form LC3-II results in the recruitment of ubiquitinated p62, also called sequestosome 1 (SQSTM1), for degradation [20]. Hence, LC3-II and p62 are generally considered as indicators of macroautophagy [24,25,26]. Autophagy-related proteins (ATGs) are key participants in this strictly regulated process [20]. In particular, ATG5 has been shown to be indispensable in autophagy [27]. For example, in response to starvation or rapamycin treatment, ATG5 binds receptor-activated C-kinase 1 (RACK1), a scaffold protein, to initiate autophagosome formation [28]. Recent studies have documented that knockout of ATG5 in mice causes neonatal lethality, possibly by disrupting autophagy and thus inhibiting the engulfment of lipid droplets [29,30,31]. Our group has demonstrated that Cd evokes impaired autophagic flux contributing to autophagosomes-dependent neurotoxicity [32]. This prompted us to further study the relationship between ATG5 and apoptosis in neuronal cells induced by Cd, as well as the underlying mechanisms.

Metformin, a well-known antidiabetic drug for patients with type 2 diabetes mellitus, has been shown to have neuroprotective effects in the models of neurodegenerative disorders [33,34,35]. Of the various potential targets of metformin identified, AMP-activated protein kinase (AMPK), a master controller of metabolic homeostasis, has been placed at central stage [36,37,38]. Studies have shown that AMPK, through phosphorylation of acetyl coenzyme A carboxylase 1/2 (ACC1/2), is essential for metformin-reducing hepatic steatosis and atherosclerosis in diabetic mice [39,40]. Metformin enhances autophagy via activating AMPK in diabetic kidney disease, pulmonary fibrosis and other diseases [41,42]. Our recent studies have identified that metformin attenuates Cd-induced apoptosis via blocking the ROS-dependent PP5/AMPK-JNK signaling pathway in neuronal cells [43]. Based on these findings, we postulated that metformin may also play a protective role by activating AMPK to regulate autophagy in Cd-induced neuronal cell death.

Here, for the first time, we show that metformin protects neuronal cells from Cd-induced autophagic flux impairment-dependent apoptosis by activating AMPK. The results improve our understanding of the molecular mechanism by which metformin has a potential for the prevention of Cd-induced autophagy dysfunction and toxicity related to neurodegenerative diseases.

## 2. Materials and Methods

### 2.1. Reagents

Cadmium chloride, metformin, chloroquine diphosphate (CQ), monodansylcadaverine (MDC), poly-D-lysine (PDL), 4′,6-diamidino-2-phenylindole (DAPI), and a protease inhibitor cocktail were sourced from Sigma-Aldrich (St. Louis, MO, USA). The AMPK activator, 5-amino-4-imidazolecarboxamide ribose (AICAR), was obtained from Enzo Life Sciences (Farmingdale, NY, USA). For cell viability assessments, the CellTiter 96^®^ AQ_ueous_ One Solution Cell Proliferation Assay kit was purchased from Promega (Madison, WI, USA). Chemiluminescent detection was facilitated using an enhanced chemiluminescence solution from Sciben Biotech (Nanjing, China). Cell culture reagents, including Dulbecco’s Modified Eagle Medium (DMEM), 0.05% Trypsin-EDTA, NEUROBASAL™ Media, and B-27 Supplement, were acquired from Invitrogen (Grand Island, NY, USA). Fetal bovine serum (FBS) and horse serum were supplied by Hyclone (Logan, UT, USA). All other chemicals, procured from local suppliers, were of analytical grade to ensure experimental rigor.

### 2.2. Cell Culture

The rat pheochromocytoma (PC12) cell line, a widely utilized neuronal model, was obtained from the American Type Culture Collection (ATCC, Manassas, VA, USA). For experimental procedures, PC12 cells were seeded onto culture vessels (6-well or 96-well plates) pre-coated with PDL (0.2 μg/mL) to promote adhesion. Cells were maintained in antibiotic-free DMEM supplemented with 10% horse serum and 5% FBS within a humidified incubator at 37 °C with 5% CO_2_. To corroborate findings obtained from the PC12 cell line, primary cortical neurons were isolated from the cerebral cortices of fetal ICR mice at gestational day 16–18, following a previously described protocol [44]. The isolated neurons were subsequently cultured on plates coated with PDL (10 μg/mL) and maintained under identical conditions (37 °C, 5% CO_2_) for 6 days prior to experimental manipulation. All animal procedures were conducted in strict accordance with the guidelines established by the Guide for the Care and Use of Laboratory Animals. The experimental protocol was reviewed and approved by the Institutional Animal Care and Use Committee of Nanjing Normal University (Certificate No. 200408).

### 2.3. Recombinant Adenoviral Construction and Transduction

Recombinant adenoviruses encoding a constitutively active mutant of rat AMPKα1 (T172D) tagged with myc (Ad-AMPKα-ca) [45] were kindly provided by Dr. Kenneth Walsh (Boston University School of Medicine, Boston, MA, USA). A control adenovirus expressing β-galactosidase (Ad-LacZ) was generated as previously characterized [46]. Additionally, adenoviruses harboring GFP-tagged LC3 (Ad-GFP-LC3) or a tandem mCherry-GFP-LC3 fusion construct (Ad-mCherry-GFP-LC3) were commercially obtained from Sciben Biotech (Nanjing, China). For experimental transduction, PC12 cells were cultured in standard growth medium and subsequently infected with the respective adenoviruses at a multiplicity of infection (MOI) of 5 for 24 h. Following transduction, cells were subjected to downstream assays. Infection with Ad-LacZ served as a negative control to account for non-specific viral effects. Successful overexpression of myc-tagged AMPKα-ca was confirmed by immunoblotting using an anti-myc antibody.

### 2.4. Lentiviral-Mediated Gene Silencing

Lentiviral constructs encoding short hairpin RNAs (shRNAs) targeting ATG5, as well as a non-targeting control shRNA against GFP, were generated and produced following established protocols [47]. For transduction, PC12 cells were cultured until approximately 70% confluence and then exposed to lentivirus-containing supernatants supplemented with polybrene (8 μg/mL) to enhance infection efficiency. The infection procedure was performed twice, each lasting 12 h, with a 6 h interval between rounds. Following transduction, successfully infected cells were selected by culturing in the presence of puromycin (2 μg/mL) for 48 h to eliminate non-transduced populations. After an additional 5 days of culture to allow for adequate gene silencing, the cells were harvested for subsequent experiments.

### 2.5. Assessment of Cell Viability

Cell viability was evaluated using the MTS colorimetric assay. PC12 cells or primary murine neurons were plated in PDL-coated 96-well plates at a density of 1 × 10^4^ cells per well and maintained under standard culture conditions (37 °C, 5% CO_2_). Following overnight attachment, cells were pre-incubated with varying concentrations of metformin (0.2, 0.5, 1, and 1.5 mM) for 24 h, followed by exposure to Cd (10 μM) for an additional 24 h. Subsequently, 20 μL of MTS reagent (CellTiter 96^®^ AQueous One Solution) was added to each well, and the plates were incubated for 3 h at 37 °C. The optical density (OD) was measured at 490 nm using a Victor X3 multilabel plate reader (PerkinElmer, Waltham, MA, USA). All experiments were performed in five replicates to ensure reproducibility.

### 2.6. DAPI and TUNEL Staining

Apoptotic cell death was evaluated through morphological nuclear staining and DNA fragmentation analysis. PC12 cells, primary neurons, and genetically modified PC12 variants (including cells transduced with lentiviral shRNA targeting ATG5 or control shRNA against GFP, as well as those infected with Ad-AMPKα-ca or Ad-LacZ) were seeded onto PDL-coated glass coverslips in 6-well plates at a density of 5 × 10^5^ cells per well. Following overnight adhesion, cells were subjected to various treatment regimens. These included pre-incubation with/without metformin (0.2–1.5 mM or 1 mM) for 24 h, with/without subsequent exposure to the autophagic flux inhibitor CQ (25 μM) [32] or the AMPK activator AICAR (2 mM) [43] for 1 h, followed by treatment with/without Cd (10 μM) for an additional 24 h. Each experimental condition was performed in five replicates. For nuclear morphology assessment, cells were stained with DAPI to visualize fragmented and condensed apoptotic nuclei as described [46]. To detect DNA strand breaks, terminal deoxynucleotidyl transferase dUTP nick-end labeling (TUNEL) staining was performed using the In Situ Cell Death Detection Kit^®^ (Roche, Mannheim, Germany) according to the manufacturer’s protocol. Briefly, cells were incubated with the TUNEL reaction mixture containing TdT enzyme and labeling solution. Fluorescent images were captured using a Leica DMi8 fluorescence microscope (Wetzlar, Germany) equipped with a digital camera (200× magnification). For quantitative analysis of TUNEL staining, the integral optical density (IOD) was measured using Image-Pro Plus 6.0 software (Media Cybernetics Inc., Newburyport, MA, USA).

### 2.7. GFP-LC3 or mCherry-GFP-LC3 Assay

To monitor autophagosome formation and autophagic flux, cells infected with Ad-GFP-LC3 or Ad-mCherry-GFP-LC3, respectively, were seeded onto PDL-coated glass coverslips in 6-well plates at a density of 5 × 10^5^ cells per well. After overnight attachment and subsequent experimental treatments, cells were fixed with 4% paraformaldehyde in phosphate-buffered saline (PBS) for 30 min at 4 °C, followed by three washes with PBS. For cells expressing mCherry-GFP-LC3, nuclei were counterstained with DAPI as described [46]. All fluorescent images were acquired using a Leica DMi8 fluorescence microscope equipped with a digital camera. For cells expressing GFP-LC3 alone, autophagosome formation was quantitatively assessed by counting the number of cells exhibiting punctate GFP-LC3 fluorescence, indicative of autophagosome-associated LC3. For cells expressing the tandem mCherry-GFP-LC3 reporter, a more detailed analysis of autophagic flux was performed. This assay exploits the differential pH stability of the two fluorescent proteins: GFP fluorescence is acid-labile and quenched upon delivery to the acidic lysosomal compartment, whereas mCherry fluorescence remains relatively stable. Consequently, autophagosomes (which have not yet fused with lysosomes) appear as yellow puncta (GFP^+^/mCherry^+^) in merged images due to co-localization of both signals. Upon autophagosome-lysosome fusion and subsequent acidification, GFP fluorescence is attenuated, resulting in autolysosomes appearing as red puncta (GFP^−^/mCherry^+^). Thus, the relative abundance of yellow versus red puncta provides a readout of autophagic flux efficiency: an accumulation of yellow puncta suggests impaired autophagosome-lysosome fusion or defective lysosomal acidification, while an increase in red puncta indicates normal progression to autolysosomes. Quantification was performed by counting cells with predominant yellow puncta (representing autophagosomes) or red puncta (representing autolysosomes).

### 2.8. Detection of Autophagic Vacuoles by MDC Staining

Autophagic vacuole formation was assessed using monodansylcadaverine (MDC), a fluorescent probe widely employed for labeling autophagic compartments as described [32]. PC12 cells and primary neurons were seeded onto PDL-coated glass coverslips in 6-well plates at a density of 5 × 10^5^ cells per well. Following overnight adhesion, cells were pre-incubated with/without metformin (0.2, 0.5, 1, and 1.5 mM) for 24 h, followed by exposure to Cd (10 μM) for an additional 12 h. Each experimental condition was performed in five replicates. After treatment, cells were incubated with MDC (0.05 mM in PBS) for 10 min at 37 °C to label autophagic vacuoles. Following three washes with PBS to remove unbound dye, fluorescent images were immediately captured using a Leica DMi8 fluorescence microscope equipped with a digital camera. For quantitative analysis of MDC fluorescence intensity, the integral optical density (IOD) was measured using Image-Pro Plus 6.0 software (Media Cybernetics Inc., Newburyport, MA, USA).

### 2.9. Immunoblot Analysis

Following experimental treatments, cells were rinsed with ice-cold PBS and subsequently lysed in radioimmunoprecipitation assay buffer on ice. Protein extracts were subjected to immunoblotting as described [46]. The following primary antibodies were utilized for immunodetection: phospho-AMPKα (p-AMPKα) (Thr172), phospho-acetyl-CoA carboxylase (p-ACC) (Ser79), ACC, cleaved caspase-3, and poly(ADP-ribose) polymerase (PARP), all obtained from Cell Signaling Technology (Danvers, MA, USA); LC3, SQSTM1/p62, and ATG5 from Sigma-Aldrich (St. Louis, MO, USA); AMPKα from Epitomics (Burlingame, CA, USA); and myc epitope, β-tubulin from Sciben Biotech (Nanjing, China). Horseradish peroxidase (HRP)-conjugated secondary antibodies, including goat anti-rabbit IgG, goat anti-mouse IgG, and rabbit anti-goat IgG, were also sourced from Sciben Biotech. Immunoreactive bands were visualized using enhanced chemiluminescence detection. Densitometric analysis of the blots was performed using NIH Image J 1.37v software (National Institutes of Health, Bethesda, MD, USA), with protein expression levels normalized to β-tubulin as a loading control.

### 2.10. Data and Statistical Analysis

All data are presented as mean ± standard error of the mean (SEM). Each experiment included at least five independent biological replicates (*n* = 5), as specified in the corresponding figure legends. For Western blotting, MDC staining, GFP-LC3 puncta quantification, and apoptosis assays, each biological replicate was derived from an independently cultured batch of cells, and technical replicates (e.g., duplicate wells or triplicate measurements) were averaged to yield a single value per replicate.

Normality of data distribution was assessed using the Shapiro–Wilk test, and homogeneity of variances was evaluated using Levene’s test. For comparisons between two groups, statistical significance was determined using two-tailed Student’s *t*-test. For multiple group comparisons, one-way or two-way analysis of variance (ANOVA) was performed, followed by Bonferroni’s post hoc test for pairwise comparisons. *p* < 0.05 was considered statistically significant. To minimize bias, all experiments were performed with randomization of treatment allocation and blinded outcome assessment where feasible. Specifically, for image-based quantifications (DAPI, TUNEL, MDC, GFP-LC3, and mCherry-GFP-LC3), samples were coded, and the investigator performing the analysis was unaware of treatment conditions until data collection was complete.

## 3. Results

### 3.1. Metformin Attenuates Cd-Induced Apoptosis in Neuronal Cells

**Figure 1:**
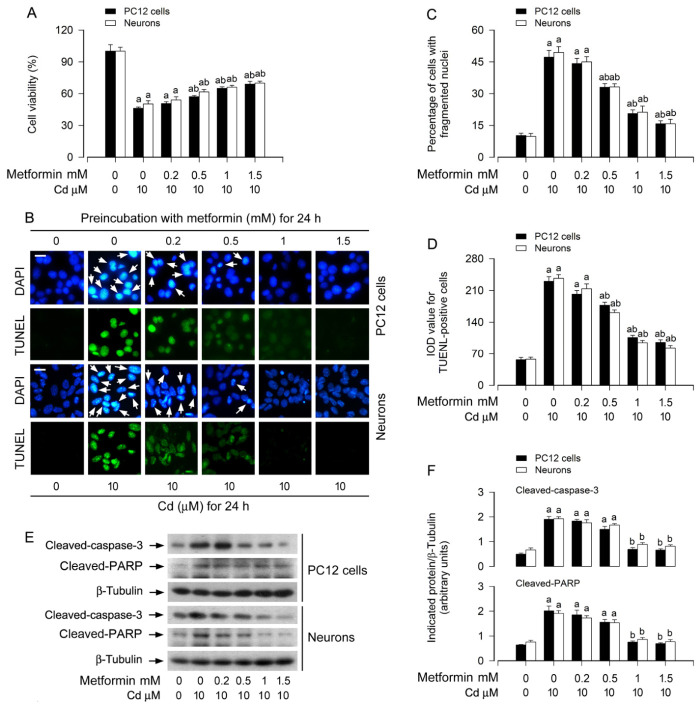
Metformin protects neuronal cells against Cd-induced apoptosis. Experimental timelines were tailored to specific assays: PC12 cells and primary neurons were pre-incubated with metformin (0–1.5 mM) for 24 h, followed by exposure to Cd (10 μM) for 4 h (for immunoblotting) or 24 h (for cell viability and apoptosis assays). (**A**) Cell viability was measured by MTS assay and expressed as a percentage relative to untreated controls. (**B**) Representative fluorescence micrographs illustrated apoptosis via DAPI staining (nuclear condensation/fragmentation, indicated by arrows, upper panels) and TUNEL (DNA strand breaks, green, lower panels). Scale bar: 20 μm. (**C**,**D**) Quantification of DAPI-positive apoptotic nuclei and TUNEL-positive cells from experiments shown in (**B**). (**E**) Whole-cell extracts were analyzed by immunoblot analysis with the specified antibodies. β-tubulin served as a loading control. Blots were representative of five independent experiments. (**F**) Densitometric analysis of cleaved caspase-3 and cleaved PARP bands normalized to β-tubulin was performed using NIH Image J software. Data are expressed as mean ± SEM (*n* = 5). ^a^ *p* < 0.05 compared to control group; ^b^ *p* < 0.05 compared to 10 μM Cd alone group.

Our recent work demonstrated that metformin confers neuroprotection against Cd toxicity through suppression of the ROS-dependent PP5/AMPK-JNK signaling axis [43]. Consistent with these observations, the present study further reveals that pretreatment with metformin (0.2–1.5 mM, 24 h) dose-dependently mitigated apoptotic cell death induced by Cd exposure (10 μM, 24 h) in both PC12 cells and primary murine neurons. This protective effect was corroborated by multiple complementary assays. First, MTS colorimetric assays revealed that cell viability was significantly higher in cultures co-treated with Cd and metformin (0.5–1.5 mM) compared to those exposed to Cd alone (Figure 1A). Second, DAPI staining demonstrated that Cd exposure markedly increased the frequency of nuclear fragmentation and condensation (arrows), a morphological hallmark of apoptosis [48], in both PC12 cells and primary neurons, an effect substantially attenuated by metformin (0.5–1.5 mM) pretreatment (Figure 1B,C). Third, TUNEL staining showed a pronounced reduction in the number of apoptotic (TUNEL-positive) cells following metformin pretreatment in Cd-exposed cultures (Figure 1B,D). Fourth, immunoblot analysis revealed that Cd treatment (4 h) robustly induced cleavage of caspase-3 and PARP in both cell types, and this effect was diminished by metformin in a concentration-dependent manner (Figure 1E,F). Collectively, these findings provide compelling evidence that metformin effectively protects neuronal cells against Cd-induced apoptosis.

### 3.2. Metformin Mitigates Cd-Induced Upregulation of ATG5/LC3-II/P62, Autophagosome Accumulation, and Apoptosis in Neuronal Cells

**Figure 2:**
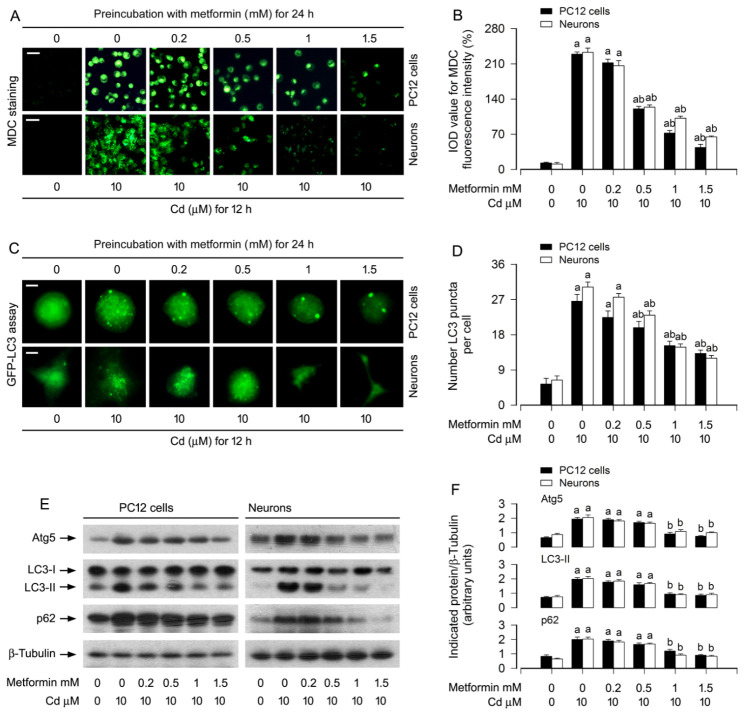
Metformin mitigates Cd-induced autophagy dysregulation in neuronal cells. PC12 cells and primary neurons, either non-infected or infected with Ad-GFP-LC3, were pre-incubated with metformin (0–1.5 mM) for 24 h, followed by exposure to Cd (10 μM) for 4 h (for Immunoblotting) or 12 h (for MDC staining and GFP-LC3 puncta analysis). (**A**,**B**) Following MDC labeling to visualize autophagic vacuoles, representative fluorescence micrographs (**A**) and quantitative analysis of MDC-positive vacuole fluorescence intensity (**B**) were presented. Scale bar: 20 μm. (**C**,**D**) Autophagosome formation was monitored via GFP-LC3 redistribution; representative images (**C**) and quantification of GFP-LC3 puncta per cell (**D**) demonstrated autophagic flux alterations. Scale bar: 2 μm. (**E**) Immunoblot analysis of autophagy-related markers was performed on whole-cell lysates using antibodies against ATG5, LC3, and p62, with β-tubulin serving as the loading control. Blots were representative of five independent experiments. (**F**) Densitometric quantification of ATG5, LC3-II, and p62 levels normalized to β-tubulin was performed using NIH Image J software. Data are expressed as mean ± SEM (*n* = 5). ^a^ *p* < 0.05 compared to control group; ^b^ *p* < 0.05 compared to 10 μM Cd alone group.

We previously reported that Cd disrupts autophagic flux via Akt pathway activation, leading to autophagosome accumulation and subsequent neuronal apoptosis [32]. Building on these findings, the present study sought to determine whether metformin exerts its neuroprotective effects by counteracting Cd-induced autophagic dysfunction. To assess autophagic activity, we first employed MDC staining, a well-established method for detecting autophagic vacuoles [49]. In PC12 cells and primary neurons, Cd exposure markedly increased MDC fluorescence intensity, indicative of autophagic vacuole accumulation. Notably, metformin pretreatment (0.2–1.5 mM) attenuated this effect in a dose-dependent manner, as evidenced by reduced fluorescence signal and quantitative analysis of MDC incorporation (Figure 2A,B). To further corroborate these observations, we performed GFP-LC3 puncta analysis, a specific assay for visualizing autophagosome formation [25]. Consistent with the MDC results, metformin dose-dependently suppressed the Cd-induced increase in GFP-LC3 puncta (green fluorescence) in both cell types (Figure 2C,D).

Autophagosome formation is critically dependent on ATG5 in canonical autophagy [27], and the conversion of LC3-I to LC3-II serves as a key indicator of autophagosome biogenesis [26]. Additionally, p62, an adaptor protein that links ubiquitinated substrates to LC3, is commonly used as a marker of autophagic degradation, with its accumulation reflecting impaired autophagic flux [50]. To elucidate the molecular basis of our morphological findings, we examined the protein levels of ATG5, LC3-II, and p62 by immunoblotting. The results demonstrated that metformin dose-dependently attenuated Cd-induced upregulation of all three markers in PC12 cells and primary neurons (Figure 2E,F). Collectively, these data suggest that metformin alleviates Cd-induced autophagic flux impairment, as reflected by reduced ATG5/LC3-II/p62 expression and autophagosome accumulation.

**Figure 3:**
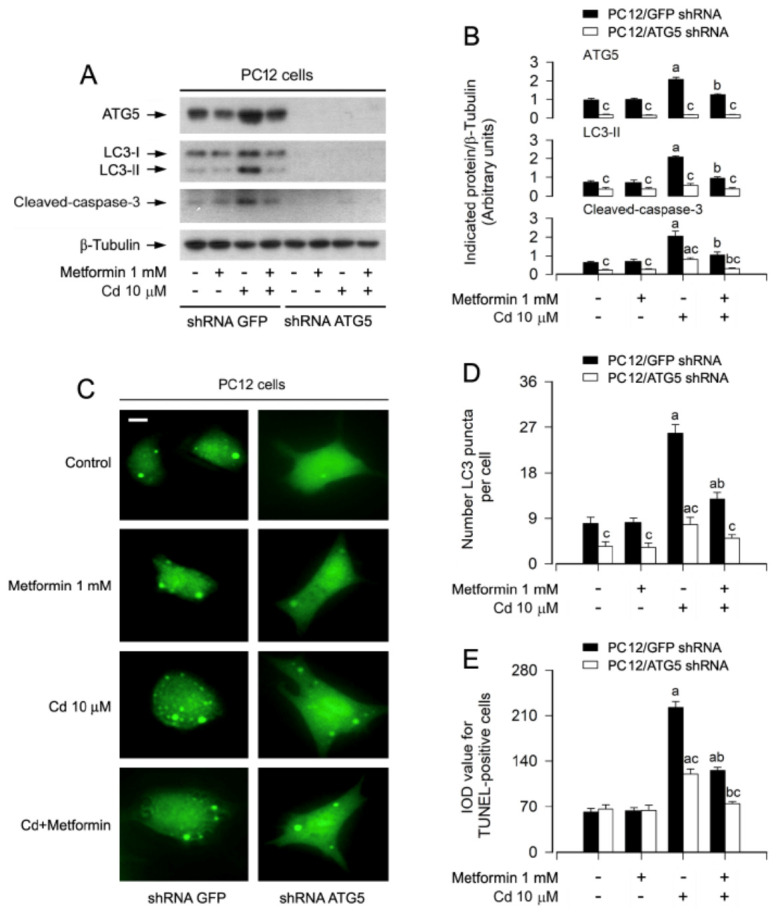
Downregulation of ATG5 augments metformin’s suppression of Cd-induced autophagosome formation and apoptosis in neuronal cells. PC12 cells were transduced with lentiviral particles encoding shRNA targeting ATG5 or a non-targeting control (GFP shRNA). Following transduction, cells were optionally infected with Ad-GFP-LC3 for subsequent autophagosome visualization, then subjected to metformin pretreatment (1 mM) for 24 h and Cd (10 μM) challenge for 4 h (for immunoblotting), 12 h (for GFP-LC3 puncta analysis), or 24 h (for TUNEL staining). (**A**) Whole-cell extracts were analyzed by immunoblot analysis with the specified antibodies. β-tubulin served as a loading control. Blots were representative of five independent experiments. (**B**) Densitometric quantification of ATG5, LC3-II, and cleaved caspase-3 levels normalized to β-tubulin was performed using NIH Image J software. (**C**) Representative fluorescence micrographs depicted the distribution of GFP-LC3 puncta (green) in the cells. Scale bar: 20 μm. (**D**) Quantitative analysis of autophagosome abundance, expressed as the number of GFP-LC3 puncta per cell, reflected the net effect of ATG5 knockdown on autophagic vacuole accumulation. (**E**) Quantification of apoptotic cells was shown via TUNEL staining, visualizing nuclear DNA strand breaks. Data are expressed as mean ± SEM (*n* = 5). ^a^ *p* < 0.05 compared to control group; ^b^ *p* < 0.05 compared to 10 μM Cd alone group. ^c^ *p* < 0.05 ATG5 shRNA group versus GFP shRNA group.

Given the essential role of ATG5 in autophagosome formation and the complete inhibition of autophagy upon its deletion [27], we next investigated whether ATG5 mediates the protective effects of metformin against Cd-induced apoptosis. To this end, ATG5 expression was silenced in PC12 cells using lentiviral shRNA, achieving approximately 90% knockdown efficiency (Figure 3A,B). As anticipated, ATG5 knockdown markedly enhanced the inhibitory effects of metformin on Cd-induced LC3-II accumulation, caspase-3 cleavage, autophagosome formation, and apoptotic cell death (Figure 3A–E). These findings underscore that metformin counteracts Cd-elicited autophagic dysfunction in an ATG5-dependent manner, thereby suppressing neuronal apoptosis.

### 3.3. Metformin Alleviates Cd-Induced Apoptosis by Restoring Impaired Autophagic Flux in Neuronal Cells

**Figure 4:**
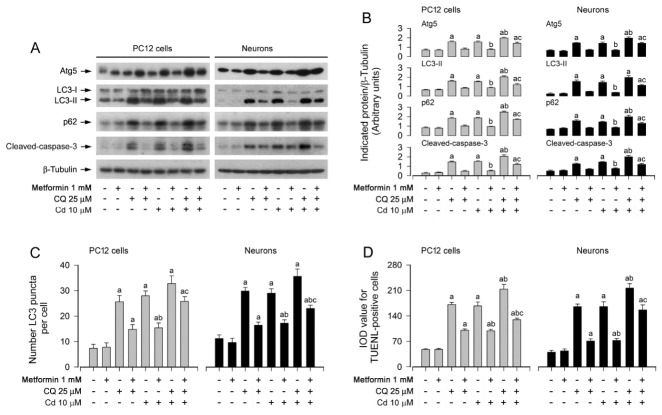
Metformin alleviates Cd-triggered autophagic flux impairment and attenuates apoptosis resulting from autophagosome accumulation in neuronal cells. PC12 cells and primary neurons, either infected with Ad-GFP-LC3 or left uninfected, were pre-incubated with/without CQ (25 μM) for 1 h, followed by metformin (1 mM) pretreatment for 24 h, and subsequently exposed in the presence or absence of Cd (10 μM) for specified durations—4 h for immunoblot analysis, 12 h for evaluation of GFP-LC3 puncta, or 24 h for detection of DNA fragmentation via TUNEL staining. (**A**) Immunoblotting of whole-cell extracts using antibodies against ATG5, LC3, p62, cleaved caspase-3, and β-tubulin (loading control). Blots were representative of five independent experiments. (**B**) Densitometric quantification of ATG5, LC3-II, p62, and cleaved caspase-3 levels normalized to β-tubulin was performed using NIH Image J software. (**C**) Quantitative analysis of autophagosome abundance was expressed as the number of GFP-LC3 puncta per cell. (**D**) Quantification of apoptotic cells was shown via TUNEL staining, visualizing nuclear DNA strand breaks. Data are expressed as mean ± SEM (*n* = 5). ^a^ *p* < 0.05 compared to control group; ^b^ *p* < 0.05 compared to 10 μM Cd alone group. ^c^ *p* < 0.05 compared to Cd/Metformin or Cd/CQ co-treatment group.

Impaired autophagic flux has been increasingly recognized as a contributing factor to cell death under various pathological conditions [51,52]. In the present study, Cd exposure resulted in a concurrent elevation of both LC3-II and p62 protein levels in neuronal cells (Figure 2). While increased LC3-II is commonly interpreted as enhanced autophagosome formation, the simultaneous accumulation of p62 suggests a disruption of autophagic flux, as effective autophagic degradation is characterized by p62 turnover rather than its sustained elevation [53,54]. This expression profile aligns with earlier reports by Tuffour et al. [55], demonstrating that Cd impairs late-stage autophagy, resulting in the accumulation of autophagosomes and cargo proteins such as p62, despite an initial increase in autophagosome biogenesis. Notably, co-treatment with metformin partially reversed the Cd-induced upregulation of both LC3-II and p62, pointing to a restoration of functional autophagic flux. Given that elevated LC3-II and p62 levels are established indicators of impaired autophagic flux [24,32], and given our observation that metformin attenuated these Cd-induced changes (Figure 2), we next sought to determine whether metformin’s protective effect against Cd-induced apoptosis is mediated through restoration of autophagic flux. To address this, we employed CQ, a lysosomotropic agent known to inhibit autophagosome-lysosome fusion, thereby blocking autophagic flux [32]. PC12 cells and primary neurons were pretreated with CQ (25 μM) for 1 h, either alone or in combination with metformin (1 mM) for 24 h, followed by exposure to Cd (10 μM) for 4 h, 12 h, or 24 h. As predicted, CQ pretreatment potentiated both basal and Cd-induced increases in ATG5/LC3-II/p62 levels (Figure 4A,B), enhanced autophagosome accumulation (Figure 4C), and exacerbated apoptotic cell death (Figure 4D). Importantly, metformin treatment robustly counteracted these CQ-aggravated effects, significantly reducing autophagic marker expression, autophagosome accumulation, and apoptosis (Figure 4A–D). The results disclose that metformin rescues neuronal cells from Cd-induced apoptosis by alleviating autophagic flux impairment. The data further underscore that the integrity of autophagic flux, restored by metformin, is essential for its neuroprotective action against Cd toxicity.

### 3.4. Metformin Counteracts Cd-Induced Autophagic Flux Impairment and Apoptosis via Activating AMPK in Neuronal Cells

**Figure 5:**
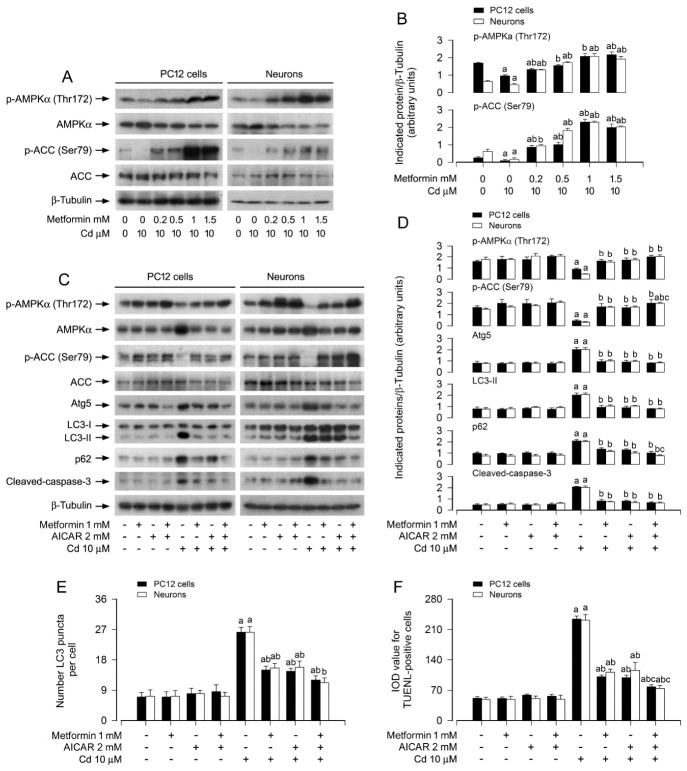
Metformin sustains autophagic flux and attenuates Cd-elicited apoptosis through reactivation of the AMPK signaling axis. PC12 cells and primary neurons, with/without Ad-GFP-LC3 infection, were pretreated with metformin (0–1.5 mM) for 24 h, or pretreated with AICAR (2 mM, 1 h) followed by metformin (1 mM), then exposed to Cd (10 μM) for 4 h (for Immunoblotting), 12 h (for GFP-LC3 puncta analysis) or 24 h (for TUNEL staining). (**A**,**C**) Whole-cell extracts were analyzed by immunoblot analysis with the specified antibodies. β-tubulin served as a loading control. Blots were representative of five independent experiments. (**B**,**D**) Densitometric quantification of p-AMPKα, p-ACC, ATG5, LC3-II, p62, and cleaved caspase-3 levels normalized to β-tubulin was performed using NIH Image J software. (**E**) Quantitative analysis of autophagosome abundance was expressed as the number of GFP-LC3 puncta per cell. (**F**) Quantification of apoptotic cells was shown via TUNEL staining, visualizing nuclear DNA strand breaks. Data are expressed as mean ± SEM (*n* = 5). ^a^ *p* < 0.05 compared to control group; ^b^ *p* < 0.05 compared to 10 μM Cd alone group. ^c^ *p* < 0.05 compared to Cd/Metformin or Cd/AICAR co-treatment group.

Metformin is a well-characterized activator of AMPK, a master regulator of cellular energy homeostasis that has been implicated in the modulation of autophagy and cell survival across multiple cell types [33,56]. Based on this, we hypothesized that AMPK activation may contribute to metformin’s protective effects against Cd-induced autophagic dysfunction and neuronal apoptosis. To test this hypothesis, we firstly examined the phosphorylation status of AMPKα (Thr172) and its downstream substrate ACC (Ser79) in PC12 cells and primary neurons following Cd exposure. Immunoblot analysis revealed that Cd treatment suppressed the phosphorylation of both AMPKα and ACC, indicating inhibition of AMPK signaling. Notably, metformin pretreatment reversed this effect in a dose-dependent manner (Figure 5A,B).

We next employed AICAR, a pharmacological AMPK activator, to determine whether AMPK activation is sufficient to phenocopy metformin’s protective effects. PC12 cells and primary neurons were pretreated with/without AICAR (2 mM) for 1 h, with/without subsequent metformin (1 mM) exposure for 24 h, followed by Cd challenge (10 μM) for 4 h or 24 h. As shown in Figure 5C,D, AICAR alone partially restored Cd-suppressed AMPKα and ACC phosphorylation, while concomitantly reducing Cd-induced upregulation of ATG5/LC3-II/p62 and cleaved-caspase-3. Strikingly, co-treatment with AICAR and metformin synergistically enhanced these inhibitory effects compared to either agent alone (Figure 5C,D). Consistent with these biochemical findings, combined AICAR and metformin treatment more potently attenuated Cd-induced autophagosome accumulation and apoptosis than monotherapy (Figure 5E,F).

**Figure 6:**
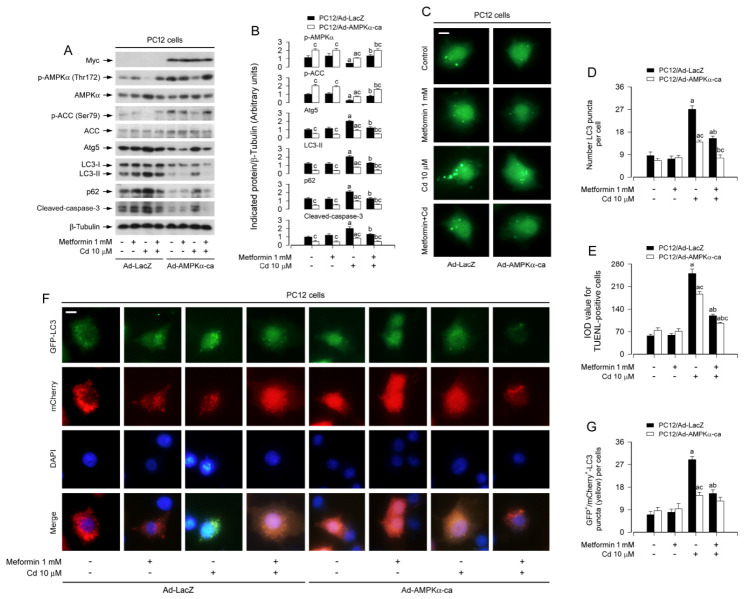
Constitutive AMPKα activation strengthens metformin’s alleviation of Cd-induced autophagic flux impairment-dependent apoptosis in neuronal cells. PC12 cells were engineered to express a constitutively active AMPKα mutant (Ad-AMPKα-ca) or GFP control (Ad-GFP), with/without subsequent Ad-GFP-LC3 or Ad-mCherry-GFP-LC3 infection for autophagic flux visualization. Cells were pretreated with/without metformin (1 mM) for 24 h, followed by exposure to Cd (10 μM) for 4 h (for immunoblotting), 12 h (for GFP-LC3 puncta and mCherry-GFP-LC3 tandem reporter assays), or 24 h (for TUNEL staining). (**A**) Whole-cell extracts were analyzed by immunoblot analysis with the specified antibodies. β-tubulin served as a loading control. Blots were representative of five independent experiments. (**B**) Densitometric quantification of p-AMPKα, p-ACC, ATG5, LC3-II, p62, and cleaved caspase-3 levels normalized to β-tubulin was performed using NIH Image J software. (**C**) Representative fluorescence micrographs depicted the distribution of GFP-LC3 puncta (green) in the cells. Scale bar: 20 μm. (**D**) Quantitative analysis of autophagosome abundance was expressed as the number of GFP-LC3 puncta per cell. (**E**) Quantification of apoptotic cells was shown via TUNEL staining, visualizing nuclear DNA strand breaks. (**F**) Representative images of tandem fluorescent mCherry-GFP-LC3 reporters: GFP signal (green) quenched in acidic lysosomal compartments, while mCherry signal (red) remains stable. Co-localized GFP^+^/mCherry^+^-LC3 (yellow in merge) puncta indicate autophagosomes that have not undergone lysosomal fusion. Scale bar: 2 μm. (**G**) Quantitative analysis of autophagic flux status was presented as the mean number of yellow (GFP^+^/mCherry^+^-LC3) puncta per cell. Data are expressed as mean ± SEM (*n* = 5). ^a^ *p* < 0.05 compared to control group; ^b^ *p* < 0.05 compared to 10 μM Cd alone group. ^c^ *p* < 0.05 Ad-AMPKα-ca group versus Ad-GFP group.

To further validate the role of AMPK, we employed a genetic gain-of-function approach. PC12 cells were transduced with recombinant adenoviruses encoding either a constitutively active mutant of AMPKα (Ad-AMPKα-ca) or a control β-galactosidase (Ad-LacZ), followed by Cd exposure with/without metformin pretreatment. Successful overexpression of myc-tagged AMPKα-ca was confirmed by immunoblotting (Figure 6A,B). Ectopic expression of AMPKα-ca markedly enhanced basal and Cd-suppressed AMPK activity, as reflected by increased phosphorylation of AMPKα and ACC (Figure 6A,B). Importantly, AMPKα-ca expression significantly attenuated Cd-induced increases in ATG5/LC3-II/p62, cleaved-caspase-3, autophagosomes and apoptosis, and these effects were further potentiated by metformin co-treatment (Figure 6A–E). Moreover, tandem mCherry-GFP-LC3 reporter assays revealed that AMPKα-ca expression enhanced metformin’s ability to reduce Cd-induced accumulation of yellow (GFP^+^/mCherry^+^) puncta, indicative of restored autophagic flux (Figure 6F,G). Taken together, these pharmacological and genetic rescue experiments demonstrate that AMPK activation is both necessary and sufficient for metformin’s protective effects against Cd-induced autophagic flux impairment and neuronal apoptosis. The data establish AMPK as a critical nodal point through which metformin counteracts Cd neurotoxicity.

## 4. Discussion

Because of its high rates of soil-to-plant transfer, Cd is a contaminant found in most human foodstuffs, which renders diet a primary source of exposure among nonsmoking, nonoccupationally exposed populations [5]. Accumulating evidence links environmental exposure to Cd with increased incidence of a variety of human diseases, including neurodegenerative diseases (PD, AD, HD and ALS) [3,6,12,13,14]. Our group has shown that Cd evokes increases in LC3-II/p62/autophagosomes contributing to apoptosis via impairing autophagic flux in neuronal cells [32]. Therefore, it is of great importance to find an effective intervention to prevent Cd-induced autophagy dysfunction and neurotoxicity. Metformin, a well-known antidiabetic drug, has been proposed to possess neuroprotective activity in the models of neurodegenerative disorders [33,34,35,57]. Our recent studies have demonstrated that metformin ameliorates Cd neurotoxicity via blocking ROS-dependent PP5/AMPK-JNK signaling pathway [43]. However, little is known about whether and how metformin can reverse Cd-induced impaired autophagy and apoptosis in neuronal cells. Here, for the first time, we provide evidence that metformin inhibits Cd-induced autophagic flux impairment-dependent apoptosis by preventing Cd deactivation of AMPKα in neuronal cells.

In the current study, we firstly showed that metformin attenuated Cd-induced cell viability reduction and apoptosis in PC12 cells and primary neurons dose-dependently (Figure 1A–F). Since metformin reinforces autophagy in diabetic kidney disease, pulmonary fibrosis and other diseases [41,42], and our recent study has shown that Cd impairs autophagic flux, which results in accumulation of autophagosomes contributing to neuronal apoptosis [32], we next tested whether metformin suppresses Cd-induced neuronal apoptosis by hindering autophagic dysfunction. The experiments demonstrated that metformin reverses Cd-elicited increases in autophagosomes/ATG5/LC3-II and p62 dose-dependently in PC12 cells and primary neurons, as evidenced by using MDC staining, GFP-LC3 assay and immunoblotting (Figure 2), suggesting that metformin indeed counteracts Cd-induced impairment of autophagy in neuronal cells.

In this study, to elucidate whether metformin-mitigated autophagosome accumulation plays a vital role in reversing Cd-induced neuronal apoptosis, genetic approaches were utilized. We showed that knockdown of ATG5 dramatically potentiated the inhibitory effects of metformin on Cd-induced LC3-II, autophagosomes, cleavage of caspase-3 and apoptosis in PC12 cells (Figure 3A–E). These lines of data support that metformin rectifies impairment of autophagy, counteracting Cd-triggered cell apoptosis in neuronal cells.

It has been known that when autophagic flux is defected, p62 is not degraded in autolysosomes, leading to increase in p62 in the cytosol [24]. In this study, the concurrent elevation of LC3-II and p62 following Cd exposure offers critical insight into the mechanistic basis of Cd-induced neuronal toxicity, particularly with respect to autophagic dysfunction. As established by Kumar et al. [54], p62 accumulation serves as a hallmark of impaired autophagic flux, given that this adaptor protein is normally degraded upon lysosomal delivery via autophagosomes. Cd’s ability to induce both LC3-II upregulation (autophagosome formation) and p62 accumulation (failed cargo degradation) aligns with findings from Islam et al., who demonstrated that heavy metals can compromise lysosomal integrity or impede autophagosome–lysosome fusion, thereby disrupting autophagic completion [53]. Such dysregulation initiates a self-perpetuating cycle: accumulated p62 exacerbates oxidative stress and inflammatory signaling, while compromised autophagic clearance impairs the removal of damaged proteins and organelles, collectively amplifying neuronal injury. Accordingly, Tuffour et al. corroborated the disruptive effect of cadmium on autophagic flux, reporting analogous alterations in the LC3-II/p62 ratio and linking this imbalance to enhanced neuronal apoptosis [55]. Metformin’s attenuation of LC3-II and p62 levels points to an intervention at the level of autophagic flux impairment—potentially through restoration of lysosomal function or facilitation of autophagosome turnover. This aligns with the established role of metformin as a modulator of autophagy in various cellular contexts. The resultant recovery of functional autophagic flux likely underpins the neuroprotective effects of metformin, given that efficient autophagic degradation is critical for maintaining neuronal homeostasis and counteracting cadmium-induced proteotoxic stress. Further investigations are warranted to delineate the precise molecular targets through which metformin restores autophagic flux, such as its impact on lysosomal enzyme activity, autophagosome–lysosome fusion machinery, or expression of key autophagy-related proteins. To dissect the role of autophagic flux in Cd-induced neuronal apoptosis prevented by metformin, we employed an autophagic flux blocker chloroquine (CQ) [32]. The results showed that CQ could enhance the basal and Cd-induced increases in ATG5/LC3-II/p62 levels as well as increases in autophagic vacuoles and apoptosis in PC12 cells and primary neurons (Figure 4A–D). However, metformin powerfully blocked the events, regardless of the absence or presence of Cd (Figure 4A–D). The results suggest that the intact autophagic flux recovered by metformin is vital for its rescuing Cd-induced autophagosome accumulation-dependent apoptosis in neuronal cells.

AMPK activation/phosphorylation, as a key biochemical event, regulates a variety of metabolic processes responsible for cell survival and apoptosis, and directly or indirectly promotes autophagy, which is involved in aging and neurodegenerative diseases [56,58,59,60]. It is known that metformin, a widely accepted AMPK activator, enhances autophagy [41,42]. In this study, we observed that metformin impeded Cd-evoked decreases in p-AMPKα (Thr172) and its substrate p-ACC (Ser79) in PC12 cells and primary neurons dose-dependently (Figure 5A,B), implying that metformin prevents Cd inactivation of AMPKα, in line with our recent report [43]. Of importance, we found that activation of AMPKα with AICAR or overexpression of constitutively active AMPKα (AMPKα-ca) reinforced the inhibitory effects of metformin on Cd-evoked ATG5/LC3-II/p62/autophagosomes and apoptosis in PC12 cells and/or primary neurons (Figure 5 and Figure 6). Using mCherry-GFP tandem-tagged LC3 assay, we further demonstrated that overexpression of AMPKα-ca strengthened metformin’s diminishment of Cd-evoked GFP^+^/mCherry^+^-LC3 (yellow) puncta in the cells (Figure 6F,G). Overall, these observations support that metformin prevents Cd-impaired autophagic flux and subsequent autophagosome accumulation against Cd neurotoxicity partly by restraining Cd inactivation of AMPK.

Beyond the neuroprotective effects demonstrated in the present study, the capacity of metformin to attenuate Cd-induced toxicity raises intriguing questions regarding its broader applicability—particularly in the context of Cd-associated renal injury, a well-established manifestation of chronic Cd exposure. The kidney represents a primary target organ for Cd toxicity, owing to its central role in Cd accumulation and excretion. Prolonged exposure leads to proximal tubular damage, interstitial fibrosis, and progressive renal dysfunction [61]. Notably, the pathophysiology of Cd-induced nephrotoxicity shares key mechanistic features with the neuronal injury described herein, including oxidative stress, inflammation, and disruption of autophagic flux [54,55]. Given metformin’s well-documented renoprotective effects—attributed to its modulation of oxidative stress, autophagy, and mitochondrial homeostasis [62]—it is plausible that metformin may engage these convergent pathways to mitigate Cd-induced renal damage. For instance, metformin has been demonstrated to attenuate renal fibrosis and oxidative stress in models of diabetic nephropathy, an effect attributed to the restoration of autophagic flux—a mechanism that may similarly counteract Cd-induced autophagic dysfunction in renal tubular cells. Elucidating this pathway could position metformin as a repurposable therapeutic candidate for Cd-induced nephrotoxicity, particularly in occupational or environmental settings characterized by chronic, high-level Cd exposure. Beyond renal protection, the neuroprotective effects of metformin against Cd toxicity raise the possibility of its broader efficacy against other heavy metals with established neurotoxic potential, such as methylmercury (MeHg). MeHg is a potent environmental neurotoxin capable of crossing the blood–brain barrier, leading to neurodegeneration, cognitive impairment, and developmental neurotoxicity [63]. Notably, the mechanisms underlying MeHg-induced neurotoxicity—oxidative stress, mitochondrial dysfunction, and autophagic disruption—closely parallel those implicated in Cd toxicity and represent pathways amenable to metformin’s pharmacological modulation [53,64]. For example, MeHg has been shown to induce excessive reactive oxygen species (ROS) production and impair autophagic flux in neuronal cells, culminating in the accumulation of damaged proteins and apoptotic cell death [65]. Consistent with the findings of the present study, metformin’s capacity to attenuate oxidative stress, restore mitochondrial function, and normalize autophagic flux positions it as a candidate for counteracting MeHg-induced neurotoxicity. Moreover, metformin’s activation of the AMPK pathway—a master regulator of cellular energy homeostasis and autophagic progression—may further alleviate MeHg-triggered metabolic dysregulation in neurons [66]. Although direct evidence for metformin’s efficacy against MeHg neurotoxicity remains limited, preclinical studies have demonstrated its protective effects against neurotoxicity induced by other heavy metals (e.g., lead, arsenic) via overlapping mechanisms. Extending this line of inquiry to MeHg-exposed neuronal models could substantiate metformin’s potential as a broad-spectrum neuroprotective agent against heavy metal toxicity, with particular relevance to populations at risk of dietary MeHg exposure (e.g., through contaminated fish consumption). Furthermore, comparative investigations into the shared and distinct mechanisms underlying metformin’s protective effects across different heavy metals—such as Cd versus MeHg—may uncover conserved therapeutic targets applicable to heavy metal-induced neurodegeneration more broadly [62].

**Figure 7:**
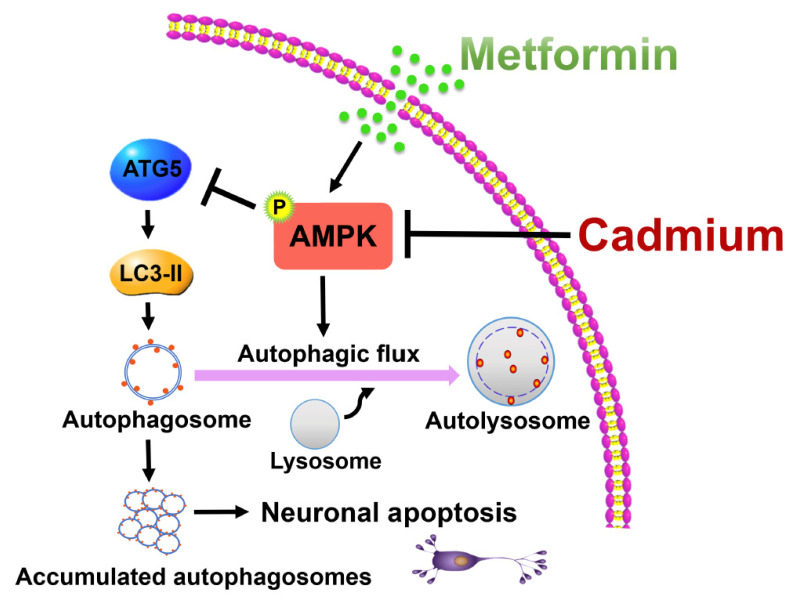
Schematic model depicting the AMPK-dependent mechanism by which metformin alleviates Cd-induced autophagic flux impairment-dependent apoptosis in neuronal cells. The illustration integrates the key molecular events: Cd exposure inactivates AMPK, leading to impaired autophagic flux and subsequent activation of apoptotic cascade. Metformin reverses this pathology by reactivating AMPK, thereby restoring autophagic degradation capacity, reducing autophagosome accumulation, and ultimately suppressing neuronal apoptosis. This model establishes autophagic flux restoration as a central mechanism underlying metformin’s therapeutic potential against heavy metal neurotoxicity.

In summary, this study demonstrates that metformin protects neuronal cells against Cd-induced apoptosis by alleviating autophagic flux impairment through an AMPK-dependent mechanism (Figure 7). These findings provide mechanistic insights into the neuroprotective action of metformin and support its potential therapeutic utility in mitigating Cd-associated neurotoxicity. Nevertheless, several limitations should be considered when interpreting the translational relevance of our work. The concentrations of metformin employed in this in vitro study (0.2–1.5 mM) exceed the clinically achievable levels typically observed in the brain and cerebrospinal fluid (low µM range). Additionally, the acute Cd exposure paradigm (10 µM for 4–24 h) does not adequately model the chronic, low-level environmental exposure more relevant to human neurodegenerative disease pathogenesis. The absence of in vivo corroboration further constrains the direct clinical applicability of our findings. Thus, while metformin emerges as a promising candidate for counteracting Cd-induced autophagic dysfunction and neurotoxicity, additional investigations are essential to ascertain its translational feasibility. Future studies should focus on: (1) in vivo pharmacokinetic analyses to determine CNS drug distribution and bioavailable concentrations in relevant brain regions; (2) utilization of chronic low-dose Cd exposure models in rodents to more closely approximate environmental exposure scenarios; (3) evaluation of metformin’s protective efficacy in neuronal subtypes particularly vulnerable to heavy metal toxicity, such as dopaminergic neurons; (4) assessment of functional neurological outcomes and behavioral correlates in animal models. Such studies are critical to establish whether the AMPK–autophagy axis identified herein represents a viable therapeutic target for preventing or ameliorating Cd-induced neurodegenerative disorders.

## Data Availability

The data supporting this manuscript can be found within the text. Any additional data and the data that supports the figures presented in this manuscript are available from the corresponding author upon reasonable request. The raw data supporting the conclusions of this article, including original immunoblotting images, are included in this published article and its Supplementary Information files.

## Supplementary Materials

The following supporting information can be downloaded at: https://www.mdpi.com/article/10.3390/cells15080739/s1, Scheme S1. Untruncated images of blots in Figure 1E; Scheme S2. Untruncated images of blots in Figure 2E; Scheme S3. Untruncated images of blots in Figure 3A; Scheme S4. Untruncated images of blots in Figure 4A; Scheme S5. Untruncated images of blots in Figure 5A,C; Scheme S6. Untruncated images of blots in Figure 6A.

## Abbreviations

ACC	acetyl-CoA carboxylase
AD	Alzheimer’s disease
AICAR	5-amino-4-imidazolecarboxamide ribose
AMPK	AMP-activated protein kinase
ATG	autophagy-related
Cd	cadmium
CQ	chloroquine diphosphate
DAPI	4′, 6-diamidino-2-phenylindole
DMEM	Dulbecco’s Modified Eagle’s Medium
FBS	fetal bovine serum
HD	Huntington’s disease
LC3	microtubule-associated protein 1 light chain 3
MDC	monodansylcadaverine
PARP	poly ADP-ribose polymerase
PBS	phosphate-buffered saline
PD	Parkinson’s disease
PDL	poly-D-lysine
TUNEL	the terminal deoxynucleotidyl transferase (TdT)-mediated deoxyuridine triphosphate (dUTP) nick-end labeling.
