# Supplementary material for: Metformin Alleviates Cadmium-Induced Autophagic Flux Impairment-Dependent Apoptosis by Activating AMPK in Neuronal Cells
Source: Cells. 2026 Apr 21;15(8):739. doi: 10.3390/cells15080739 (PMC13115256; doi:10.3390/cells15080739)
Supplement: Supplementary file 1 [file cells-15-00739-s001.zip › cells-4140588-supplementary.pdf]

## Supplementary Material

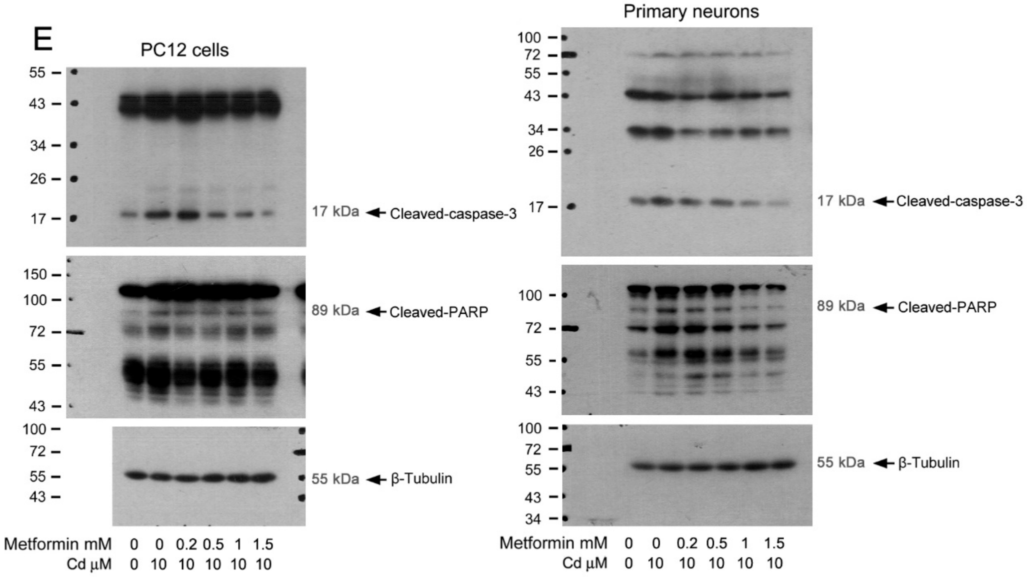


**Scheme S1. Untruncated images of blots in Figure 1E**. PC12 cells and primary neurons were pre-incubated with metformin (0–1.5 mM) for 24 h, followed by exposure to Cd (10 μM) for 4 h. Whole-cell extracts were subjected to immunoblotting using indicated antibodies. The blots were probed for β- tubulin as a loading control.

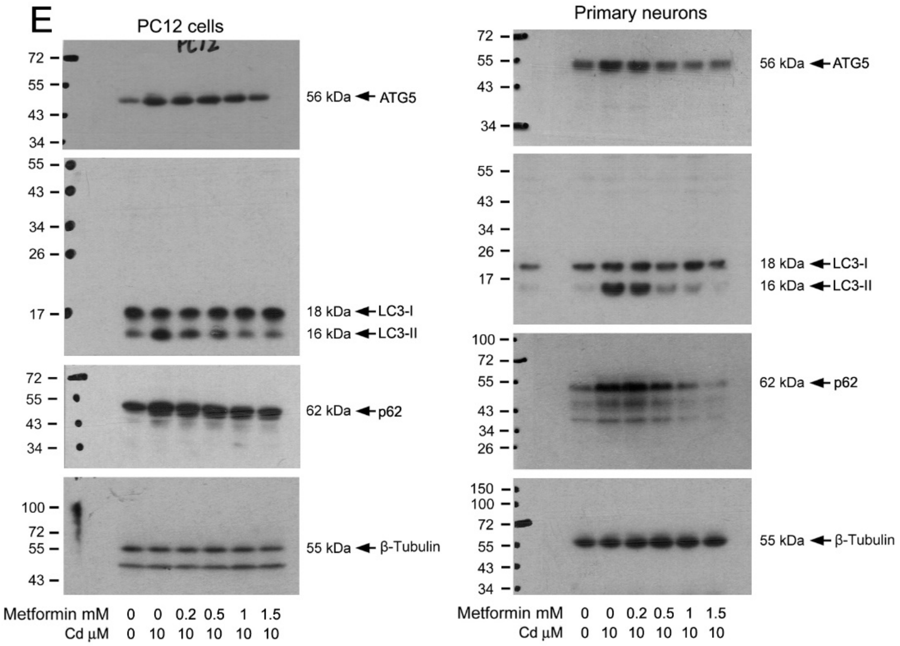


**Scheme S2. Untruncated images of blots in Figure 2E**. PC12 cells and primary neurons were pre-incubated with metformin (0–1.5 mM) for 24 h, followed by exposure to Cd (10 μM) for 4 h. Whole-cell extracts were subjected to immunoblotting using indicated antibodies. The blots were probed for β- tubulin as a loading control.

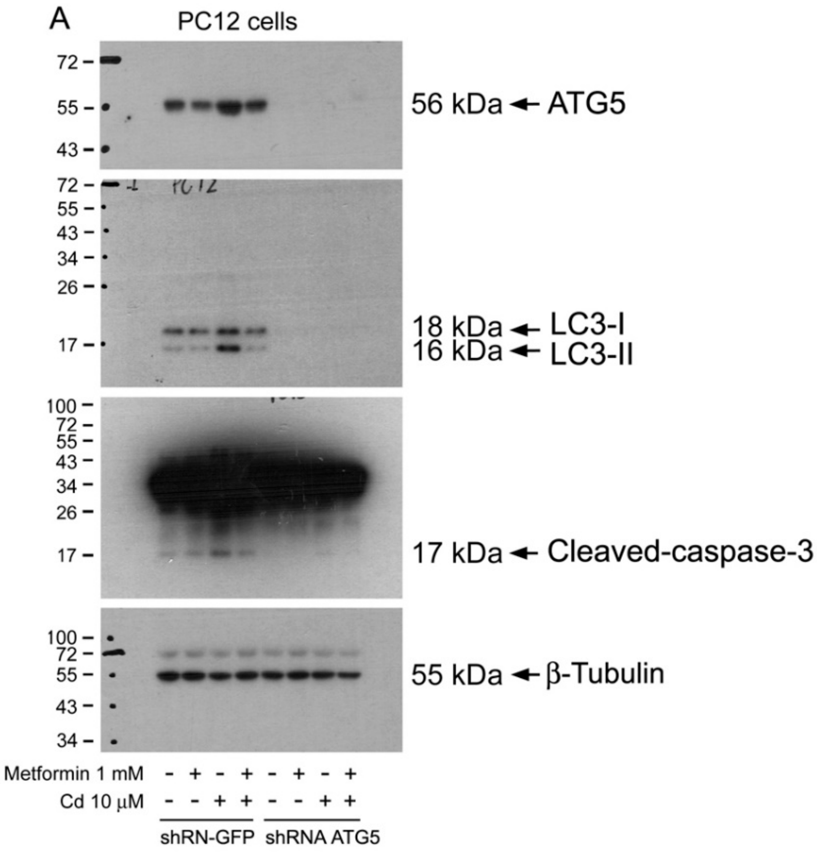


**Scheme S3. Untruncated images of blots in Figure 3A**. PC12 cells transduced with lentiviral particles encoding shRNA targeting ATG5 or a non-targeting control (GFP shRNA) were subjected to metformin pretreatment (1 mM) for 24 h and Cd (10 μM) challenge for 4 h. Whole-cell extracts were subjected to immunoblotting using indicated antibodies. The blots were probed for β-tubulin as a loading control.

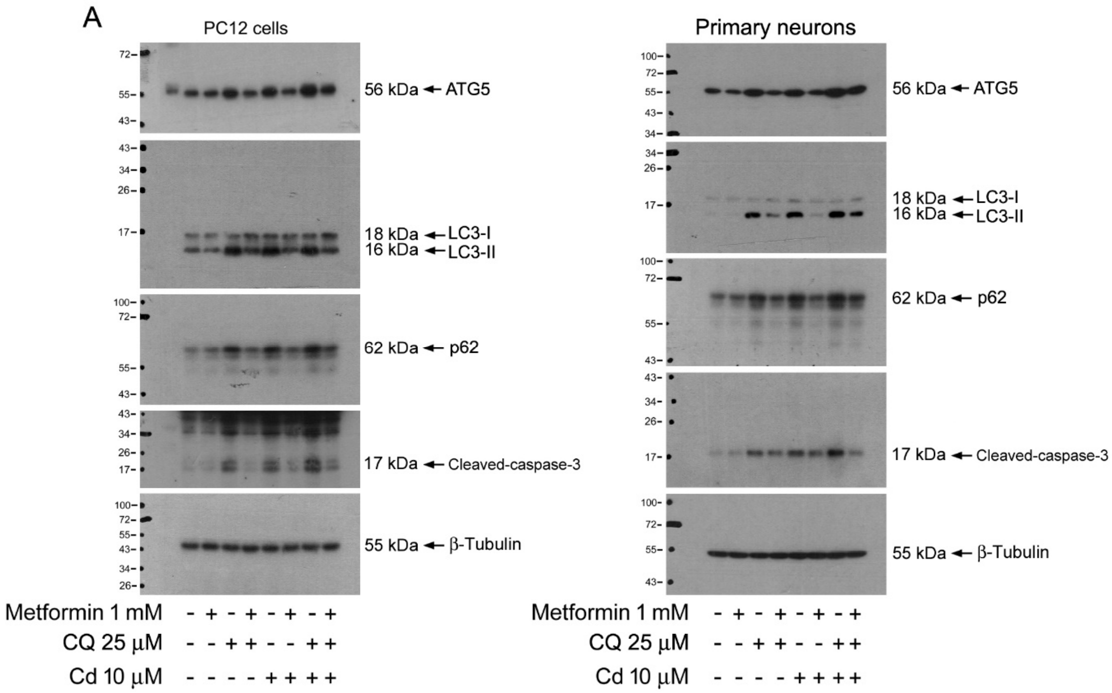


**Scheme S4. Untruncated images of blots in Figure 4A.** PC12 cells and primary neurons were pre-incubated with/without CQ (25 μM) for 1 h, followed by metformin (1 mM) pretreatment for 24 h, and subsequently exposed in the presence or absence of Cd (10 μM) for 4 h. Whole-cell extracts were subjected to immunoblotting using indicated antibodies. The blots were probed for β- tubulin as a loading control.

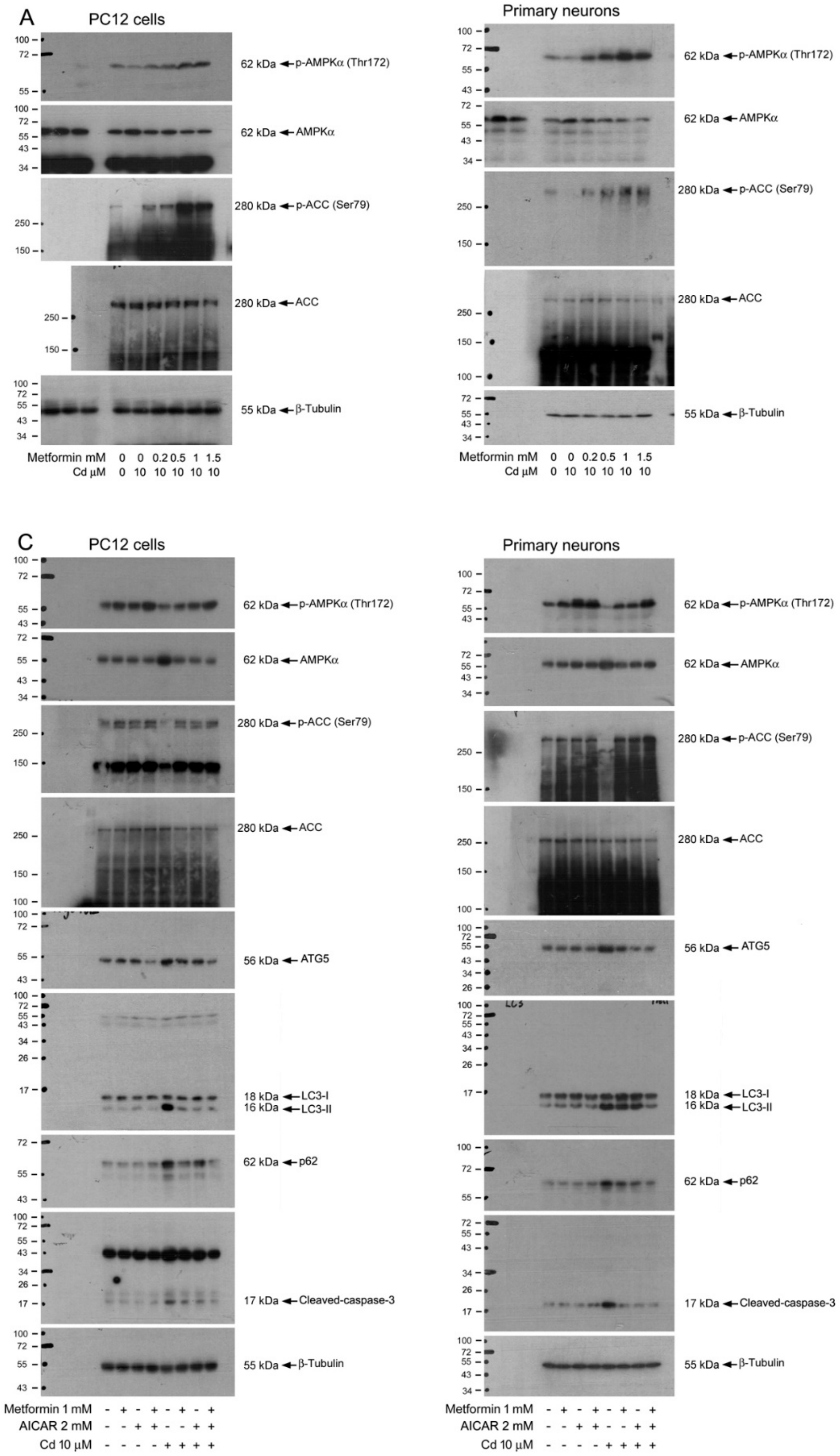


**Scheme S5. Untruncated images of blots in Figure 5A,C**. PC12 cells and primary neurons were pretreated with metformin (0-1.5 mM) for 24 h, or pretreated with AICAR (2 mM, 1 h) followed by metformin (1 mM), then exposed to Cd (10 μM) for 4 h. Whole-cell extracts were subjected to immunoblotting using indicated antibodies. The blots were probed for β-tubulin as a loading control.

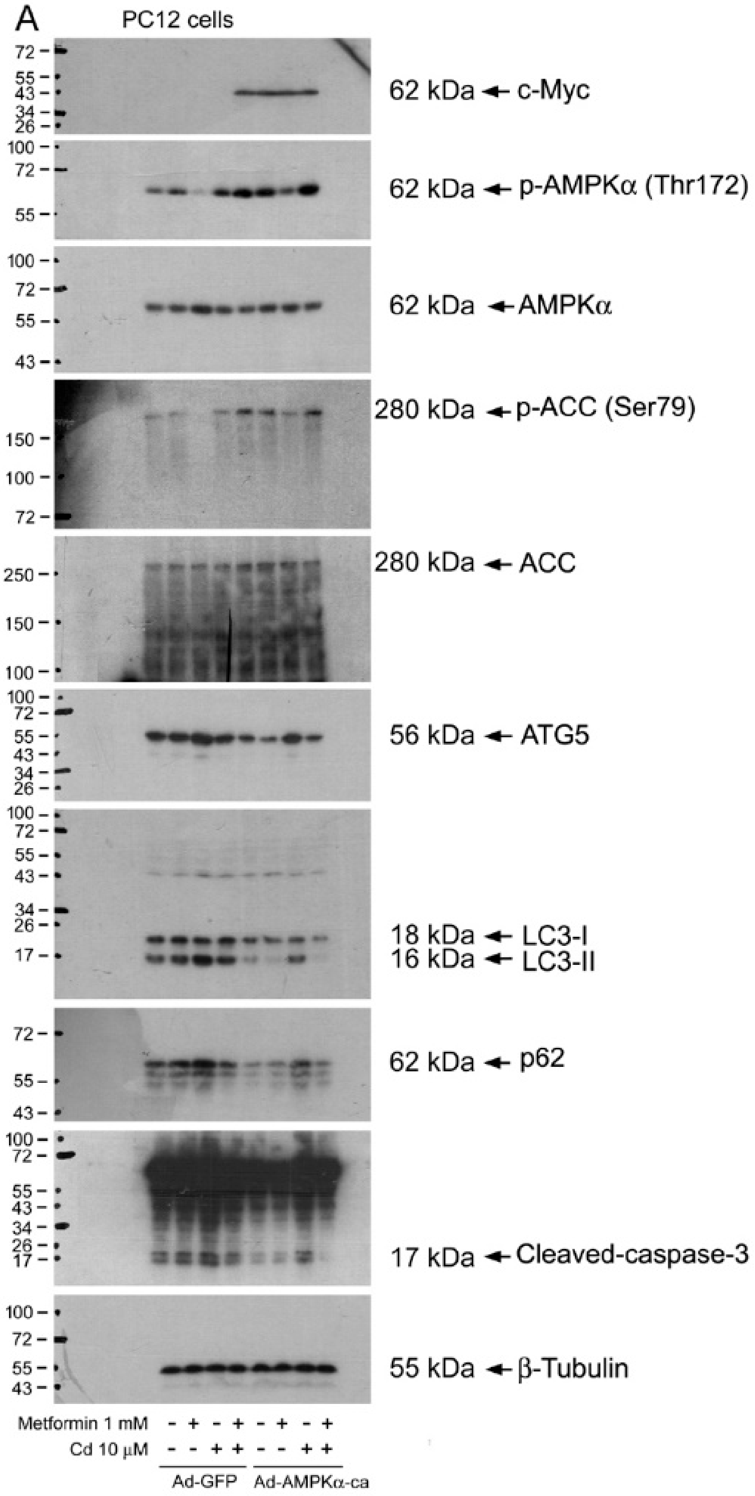


**Scheme S6. Untruncated images of blots in Figure 6A**. PC12 cells engineered to express a constitutively active AMPK$\alpha$ mutant (Ad-AMPK$\alpha$-ca) or GFP control (Ad-GFP) were pretreated with/without metformin (1 mM) for 24 h, followed by exposure to Cd (10 μM) for 4 h. Whole-cell extracts were subjected to immunoblotting using indicated antibodies. The blots were probed for β- tubulin as a loading control.
